# Preoperative combined hemoglobin, albumin, lymphocyte and platelet levels predict survival in patients with locally advanced colorectal cancer

**DOI:** 10.18632/oncotarget.12271

**Published:** 2016-09-27

**Authors:** Huihong Jiang, Huaguang Li, Ajian Li, Erjiang Tang, Dan Xu, Yin Chen, Yong Zhang, Min Tang, Zhiyong Zhang, Xiaxing Deng, Moubin Lin

**Affiliations:** ^1^ Department of General Surgery, Ruijin Hospital Affiliated to Shanghai Jiaotong University School of Medicine, Shanghai, China; ^2^ Center for Translational Medicine, Yangpu Hospital Affiliated to Shanghai Tongji University School of Medicine, Shanghai, China; ^3^ Department of General Surgery, Yangpu Hospital Affiliated to Shanghai Tongji University School of Medicine, Shanghai, China; ^4^ Department of General Surgery, Zhuji People's Hospital of Zhejiang Province, Zhejiang, China

**Keywords:** colorectal cancer, HALP, prognostic, risk, survival

## Abstract

More than 50% of patients with colorectal cancer (CRC) are initially diagnosed with locally advanced CRC (LACRC), and half of those patients develop recurrence or metastasis after resection. Here, we investigated whether the novel index HALP, which is a combination of preoperative hemoglobin, albumin, lymphocyte and platelet levels, correlates with survival in LACRC patients. A total of 820 patients with LACRC from two independent hospitals were included in our study. The correlations between HALP and overall and cancer-specific survival were calculated using training and validation sets. Lower HALP values correlated with an increased risk of death and cancer-related death in both sets. Moreover, the risk score based on HALP allowed stratification of patients into distinct prognostic groups with greater accuracy than previously proposed indexes. These results suggest that HALP may be useful as a clinical prognostic factor for patients with LACRC.

## INTRODUCTION

Colorectal cancer (CRC) is the third most common malignancy and the fourth leading cause of cancer-related death worldwide [1]. Approximately 60% of patients present locally advanced CRC (LACRC) at the initial diagnosis [2], and nearly half of them will eventually develop recurrence or metastases after curative resection [3]. Therefore, it is extremely important to identify biomarkers for selection of patients at high risk of poor survival, who may benefit from the intensive follow-up.

Systemic inflammation and nutritional status play important roles in the development and progression of various cancers including CRC [4–6]. Furthermore, a wide array of hematological parameters that reflect the immune or nutritional status of organism have been identified to be associated with cancer prognosis, such as serum albumin [7], hemoglobin [8], lymphocyte [9], neutrophil [10] and platelet [11], which are all readily available in clinical practice. Further studies revealed that combinations of these parameters, such as Onodera's prognostic nutritional index (PNI) [12], platelet-to-lymphocyte ratio (PLR) [13] and neutrophil-to-lymphocyte ratio (NLR) [14], could more accurately predict the prognosis of patients than a single index. A recent study [15] reported that a novel composite index, HALP, calculated as hemoglobin (g/L) × albumin (g/L) × lymphocytes (/L) / platelets (/L), was associated with the survival of patients with gastric carcinoma (GC). To date, there has been no study on the association of HALP with CRC survival. Here, the purpose of our study was to assess the prognostic value of HALP in patients with LACRC and to identify subgroups at high risk of poor survival.

## RESULTS

### Patient characteristics

The clinical characteristics of two independent cohorts of LACRC patients enrolled in this study are presented in Table 1. Of the 684 patients in the training set, 328 (48.0%) patients were stage II and 356 (52.0%) were stage III, with a median age of 62 years (range 21-92). The median follow-up time was 67 months. There were 231 deaths (33.8%) and 182 cancer-related deaths (26.6%), but the median OS and CSS had not been reached during the follow-up period. Among the 136 patients in the validation set, 54 (39.7%) patients had died and 46 (33.8%) patient deaths were cancer-related, with a median follow-up time of 68 months. Of all the patient characteristics analyzed, only age (*P* = 0.001), tumor location (*P* < 0.001) and differentiation grade (*P* = 0.003) displayed a difference between the training and validation sets, which were adjusted in subsequent analyses.

**Table 1:** Selected Characteristics of LACRC Patients from Two Cohorts

Characteristics	Training set (n = 684)	Validation set (n = 136)	*P* value
Age, median (range), (years)	62 (21-92)	58 (32-86)	**0.001**
Gender			
Female	288 (42.1%)	50 (36.8%)	
Male	396 (57.9%)	86 (63.2%)	0.254
Smoking history			
No	555 (81.1%)	103 (75.7%)	
Yes	129 (18.9%)	33 (24.3%)	0.157
Alcohol-drinking history			
No	558 (81.6%)	105 (77.2%)	
Yes	126 (18.4%)	31 (22.8%)	0.235
First-degree relative cancer history			
Yes	93 (13.6%)	21 (15.4%)	
No	591 (86.4%)	115 (84.6%)	0.587
Tumor location			
Rectum	335 (49.0%)	91 (66.9%)	
Colon	349 (51.0%)	45 (33.1%)	**<0.001**
Differentiation grade			
Well/moderate	472 (69.0%)	111 (81.6%)	
Poor/mucinous	212 (31.0%)	25 (18.4%)	**0.003**
Vessels/nerves invasion			
Negative	606 (88.6%)	120 (88.2%)	
Positive	78 (11.4%)	16 (11.8%)	0.883
TNM stage			
II	328 (48.0%)	70 (51.5%)	
III	356 (52.0%)	66 (48.5%)	0.511
HALP, median (range)	31.8 (2.1-128.0)	34.5 (3.6-90.4)	0.393
Death			
No	453 (66.2%)	82 (60.3%)	
Yes	231 (33.8%)	54 (39.7%)	0.200
Cancer-related death			
No	502 (73.4%)	90 (66.2%)	
Yes	182 (26.6%)	46 (33.8%)	0.094

Abbreviation: LACRC, locally advanced colorectal cancer; HALP, hemoglobin (g/L) × albumin (g/L) × lymphocytes (/L) / platelets (/L).

### Association between HALP and survival

**Figure 1:**
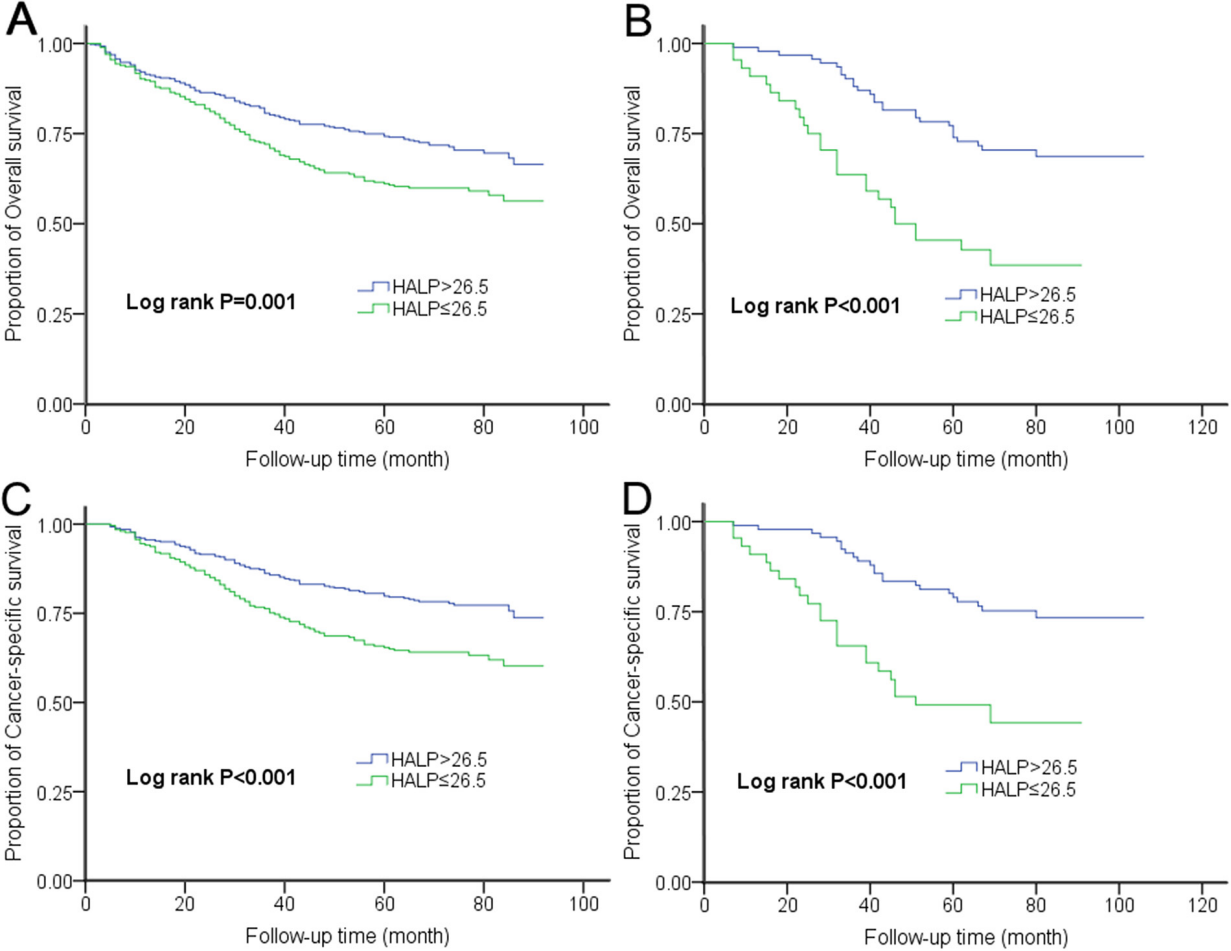
Kaplan-Meier curves for OS and CSS according to HALP in the A-C. training set and B-D. validation set

The median value of HALP was 31.8 (range 2.1-128.0) in the training set. The optimal cutoff value of HALP for both OS and CSS was computed to be 26.5 using the X-tile software (Supplementary Figure S1). Then, 684 patients were divided into low-HALP group (n = 265, 38.7%) and high-HALP group (n = 419, 61.3%). We assessed the association of HALP and some clinical variables with survival using a multivariate Cox's model. Six factors were identified to be associated with both OS and CSS after adjusting for gender, tumor location, smoking and alcohol-drinking history, including age, first-degree relative cancer history, differentiation grade, vessels/nerves invasion, TNM stage and HALP (Table 2). Patients with lower HALP exhibited an increased risk of death (HR = 1.46, 95% CI 1.11-1.92; *P* = 0.007) and cancer-related death (HR = 1.78, 95% CI 1.31-2.43; *P* < 0.001). Moreover, these patients had lower 5-year OS (60.7% vs. 74.0%; log rank *P* = 0.001) and CSS (65.0% vs. 79.6%; log rank *P* < 0.001) rates than those with higher HALP (Figure 1A and 1C). The associations between HALP and survival were confirmed in the validation set. Compared with the high-HALP group (n = 92, 67.6%), the low-HALP group (n = 44, 32.4%) showed a higher risk of death (HR = 2.38, 95% CI 1.31-4.34; *P* = 0.005) and cancer-related death (HR = 2.09, 95% CI 1.08-4.05; *P* = 0.029). The 5-year OS (45.5% vs. 73.9%; log rank *P* < 0.001) and CSS (49.1% vs. 77.8%; log rank *P* < 0.001) rates of the two groups were very different (Figure 1B and 1D).

**Table 2:** Multivariate Cox's Analyses for OS and CSS in Training Set

Variables	OS		CSS	
	HR (95% CI)	*P* value	HR (95% CI)	*P* value
Age (years)				
≤65	1 (reference)		1 (reference)	
>65	2.05 (1.57-2.68)	**<0.001**	1.99 (1.47-2.68)	**<0.001**
Gender				
Female	1 (reference)		1 (reference)	
Male	1.33 (0.99-1.76)	0.054	1.18 (0.85-1.63)	0.325
Smoking history				
No	1 (reference)		1 (reference)	
Yes	1.03 (0.68-1.56)	0.881	1.24 (0.79-1.97)	0.351
Alcohol-drinking history				
No	1 (reference)		1 (reference)	
Yes	0.85 (0.56-1.30)	0.446	0.86 (0.54-1.38)	0.531
First-degree relative cancer history				
Yes	1 (reference)		1 (reference)	
No	2.09 (1.28-3.41)	**0.003**	1.88 (1.13-3.12)	**0.016**
Tumor location				
Rectum	1 (reference)		1 (reference)	
Colon	0.89 (0.68-1.17)	0.405	0.86 (0.63-1.18)	0.352
Differentiation grade				
Well/moderate	1 (reference)		1 (reference)	
Poor/mucinous	1.69 (1.29-2.22)	**<0.001**	2.04 (1.52-2.76)	**<0.001**
Vessels/nerves invasion				
Negative	1 (reference)		1 (reference)	
Positive	1.81 (1.28-2.56)	**0.001**	1.84 (1.25-2.70)	**0.002**
TNM stage				
II	1 (reference)		1 (reference)	
III	2.07 (1.57-2.73)	**<0.001**	2.41 (1.75-3.33)	**<0.001**
HALP				
>26.5	1 (reference)			1 (reference)
≤26.5	1.46 (1.11-1.92)	**0.007**	1.78 (1.31-2.43)	**<0.001**

Abbreviation: OS, overall survival; CSS, cancer-specific survival; HR, hazard ratio; CI, confidence interval; HALP, hemoglobin (g/L) × albumin (g/L) × lymphocytes (/L) / platelets (/L).

The multivariate Cox's proportional hazard model was based on age, gender, smoking history, alcohol-drinking history, first-degree relative cancer history, tumor location, differentiation grade, vessels/nerves invasion, TNM stage and HALP.

### Association between HALP-based risk score and survival

**Figure 2:**
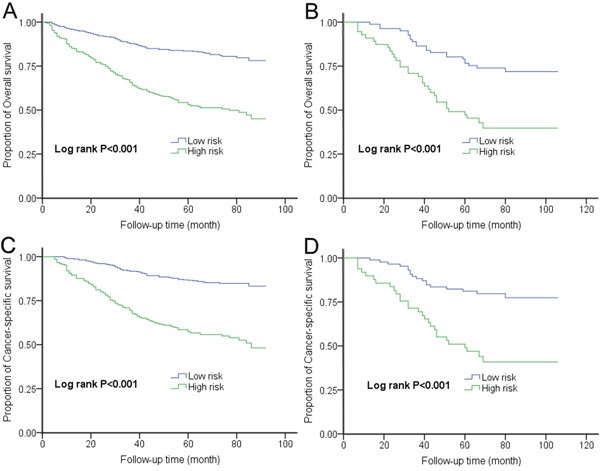
Kaplan-Meier curves for OS and CSS according to risk score in the A-C. training set and B-D. validation set

**Figure 3:**
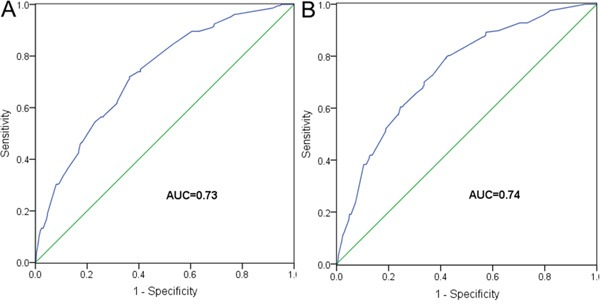
ROC analyses for A. OS and B. CSS, based on prognostic models in the training set

To further evaluate the prognostic value of HALP for survival, we performed a risk score analysis. We incorporated the six significant prognostic factors described above into a multivariate model and conducted a joint analysis. The 684 patients in the training set were categorized into low-risk and high-risk groups based on the optimal cutoff points of risk score (1.64 for OS and 1.85 for CSS) generated by X-tile analysis (Supplementary Figure S2) (Table 3). Compared with the low-risk group, the high-risk group had a 3.29-fold (95% CI 2.48-4.36; *P* < 0.001) increased risk of death and had a lower 5-year OS rate (52.5% vs. 83.6%; log rank *P* < 0.001) (Figure 2A). Moreover, the high-risk group showed a 3.87-fold (95% CI 2.82-5.30; *P* < 0.001) higher risk of cancer-related death than the low-risk group. Their respective 5-year CSS rates were also very different (56.6% vs. 86.6%; log rank *P* < 0.001) (Figure 2C). The AUCs of the prognostic models for predicting 5-year OS and CSS rates were 0.73 and 0.74, respectively (Figure 3).

**Table 3:** HALP-based risk score associated with survival in LACRC patients

Risk score	Training set			Validation set		
	No. of patients	HR (95% CI)*	*P* value	No. of patients	HR (95% CI)*	*P* value
**OS**						
Low	360 (52.6%)	1 (reference)		81 (59.6%)	1 (reference)	
High	324 (47.4%)	3.29 (2.48-4.36)	**<0.001**	55 (40.4%)	2.91 (1.69-5.03)	**<0.001**
**CSS**						
Low	393 (57.5%)	1 (reference)		87 (64.0%)	1 (reference)	
High	291 (42.5%)	3.87 (2.82-5.30)	**<0.001**	49 (36.0%)	3.57 (1.97-6.48)	**<0.001**

Abbreviation: LACRC, locally advanced colorectal cancer; OS, overall survival; CSS, cancer-specific survival; HR, hazard ratio; CI, confidence interval; HALP, hemoglobin (g/L) × albumin (g/L) × lymphocytes (/L) / platelets (/L).

* Adjusted for gender, tumor location, smoking and alcohol-drinking history.

External validation confirmed the discriminatory ability of the models (Table 3). For OS prediction, the HR for patients with higher risk scores was 2.91 (95% CI 1.69-5.03; *P* < 0.001), compared with the reference group (those with lower risk scores). This increase in death risk resulted in a decrease in OS (45.5% vs. 76.5%; log rank *P* < 0.001) (Figure 2B). Regarding CSS, patients with higher risk scores showed a 3.57 times (95% CI 1.97-6.48, *P* < 0.001) increased risk of cancer-related death than patients with lower risk scores. The 5-year CSS rates of the two groups were very different (46.9% vs. 81.1%; log rank *P* < 0.001) (Figure 2D). The AUCs were 0.72 and 0.78 for OS and CSS, respectively.

## DISCUSSION

In this study, we demonstrated that the prognostic index HALP was associated with the prognosis of LACRC patients receiving radical resection. Furthermore, the models based on HALP could effectively identify patients at high risk of poor survival.

It is widely accepted that inflammatory response and nutritional status correlates with the prognosis of cancer patients [4, 6, 16]. Serum albumin is one of the most commonly used indicators of a patient's nutritional status, and it has also been used to assess cancer progression and prognosis. Indeed, lower albumin levels correlate with poor survival of cancer patients [7, 17]. Anemia is also commonly present in various cancers, including CRC [18], and correlates with an increased risk of adverse patient outcome [8]. Lymphopenia is also frequently observed in patients with advanced cancers and stimulates cancer progression [9, 19]. Metastasis correlates with platelet stimulation [20] and platelets seem to protect cancer cells from immunological attacks [21]. HALP is the integration of these four hematological parameters, and has shown prognostic value for GC patients [15]. Our results here reveal that HALP also correlates with prognosis of LACRC patient; i.e., patients with lower HALP have worse clinical outcomes.

Various prognostic models based on hematological parameters have been proposed for CRC [22]. Kanemitsu *et al.* [23] devised a model to predict the prognosis of patients after resection of pulmonary metastases from CRC, which included preoperative carcinoembryonic antigen (CEA) level, number of pulmonary tumors, etc. The internal validated concordance index (C-index, equivalent to AUC) of this model reached 0.72, but the external validated C-index was only 0.66, suggesting this model has only moderate predictive power. Toiyama *et al.* [24] also reported a prognostic model derived from analysis of 219 patients with high-risk stage II or stage III CRC. The model was based on the Glasgow prognostic score (the combination of albumin and C-reactive protein levels). However, the C-index of such model was only 0.635. In the present study, when we incorporated six prognostic factors (including HALP) into a multivariate model, we obtained an AUC of 0.73 for OS and of 0.74 for CSS, suggesting that our model has a higher predictive ability than other models. Based on the risk score calculated by our model, we could accurately stratify patients into distinct prognostic groups, including those in the validation set. These results suggest that our model is generalizable and might be useful in clinical practice by assisting clinicians in identifying patients with poor prognosis who might benefit from more intensive follow-up and monitoring.

Our study was retrospective; therefore, the bias in data selection was unavoidable. Furthermore, analyses were limited to patients with locally advanced disease to preclude tumor stage from influencing our survival metric. Hence, further validation of our findings is warranted. Nonetheless, our study suggests that HALP is a promising prognostic factor for CRC, and that prognostic models based on HALP might present a useful tool for predicting LACRC patient survival.

## MATERIALS AND METHODS

### Study population and data collection

A total of 820 patients with histologically confirmed stage II or III colorectal adenocarcinoma were included in this study, with follow-up through January 2016. Among them, 684 patients were recruited from Ruijin Hospital affiliated to Shanghai Jiaotong University School of Medicine from January 2008 to December 2010 and were used as the training set, and 136 patients enrolled from Zhuji People's Hospital of Zhejiang Province between January 2007 and December 2010 were used as the validation set. All the patients were newly diagnosed within 3 months of enrollment and underwent radical resection. Patients who had metachronous malignancy, end-stage liver disease or chronic inflammatory disease including autoimmune disorder and infection were excluded from the study.

A series of baseline clinical variables were collected from patients’ medical records as follow: patient demographics, smoking and drinking history, family history of cancer, date of diagnosis and some tumor characteristics, such as tumor location, differentiation grade, vessels/nerves invasion and tumor stage. Patients were staged according to the 7th edition of American Joint Committee on Cancer (AJCC) TNM classification system. Moreover, four preoperative hematologic parameters including serum albumin, hemoglobin, lymphocytes and platelets were collected. Then, the HALP index was calculated as the following formula: hemoglobin (g/L) × albumin (g/L) × lymphocytes (/L) / platelets (/L) [15]. Information on vital status was obtained from medical records or telephone follow-up. This study protocol conformed to the guidelines of the ethics committee of each institution and was approved by each institution's review board.

### Statistical analysis

The endpoints of this study included overall survival (OS) and cancer-specific survival (CSS). OS was measured from the date of diagnosis till the date of death from any cause, and CSS was defined as the length of time from diagnosis to cancer-related death. Statistical analyses to identify prognostic factors were performed using SPSS software (SPSS 19.0, IBM, Chicago, IL, USA). The optimal cutoff value of HALP was determined using X-tile software (Version 3.6.1, Yale University, USA) [25]. Chi-squared and Student's t tests were used to analyze the differences in patient characteristics. To assess the association of HALP with CRC survival, multivariate Cox's proportional hazard model was conducted to estimate Hazard ratios (HRs) and their 95% confidence intervals (CIs). The Kaplan-Meier method was used to plot OS and CSS curves with the Log rank test to compare cures. We incorporated HALP and clinical variables that exhibited significant association with survival into a multivariate model and performed a risk score analysis to evaluate their combined effects in the training set. The risk score for each patient was derived by linear combination of the product of each significant risk factor by its’ corresponding Cox regression coefficient [26]. Then, patients were further categorized into low-risk and high-risk groups based on the optimal cutoff point of risk score generated by X-tile software. To evaluate the predictive efficacy of the prognostic model, we constructed receiver operating characteristic (ROC) curves and calculated the area under ROC curve (AUC) using R software (Version 3.2.0, R Foundation for Statistical Computing) [27]. The model was confirmed in the validation set. For all analyses, a *P* value of < 0.05 was considered statistically significant.

## SUPPLEMENTARY FIGURES


