# Supplementary material for: Preoperative combined hemoglobin, albumin, lymphocyte and platelet levels predict survival in patients with locally advanced colorectal cancer
Source: Oncotarget. 2016 Sep 27;7(44):72076–83. doi: 10.18632/oncotarget.12271 (PMC5342146; doi:10.18632/oncotarget.12271)
Supplement: Supplementary file 1 [file oncotarget-07-72076-s001.pdf]

Preoperative combined hemoglobin, albumin, lymphocyte and platelet levels predict survival in patients with locally advanced colorectal cancer

SUPPLEMENTARY FIGURES

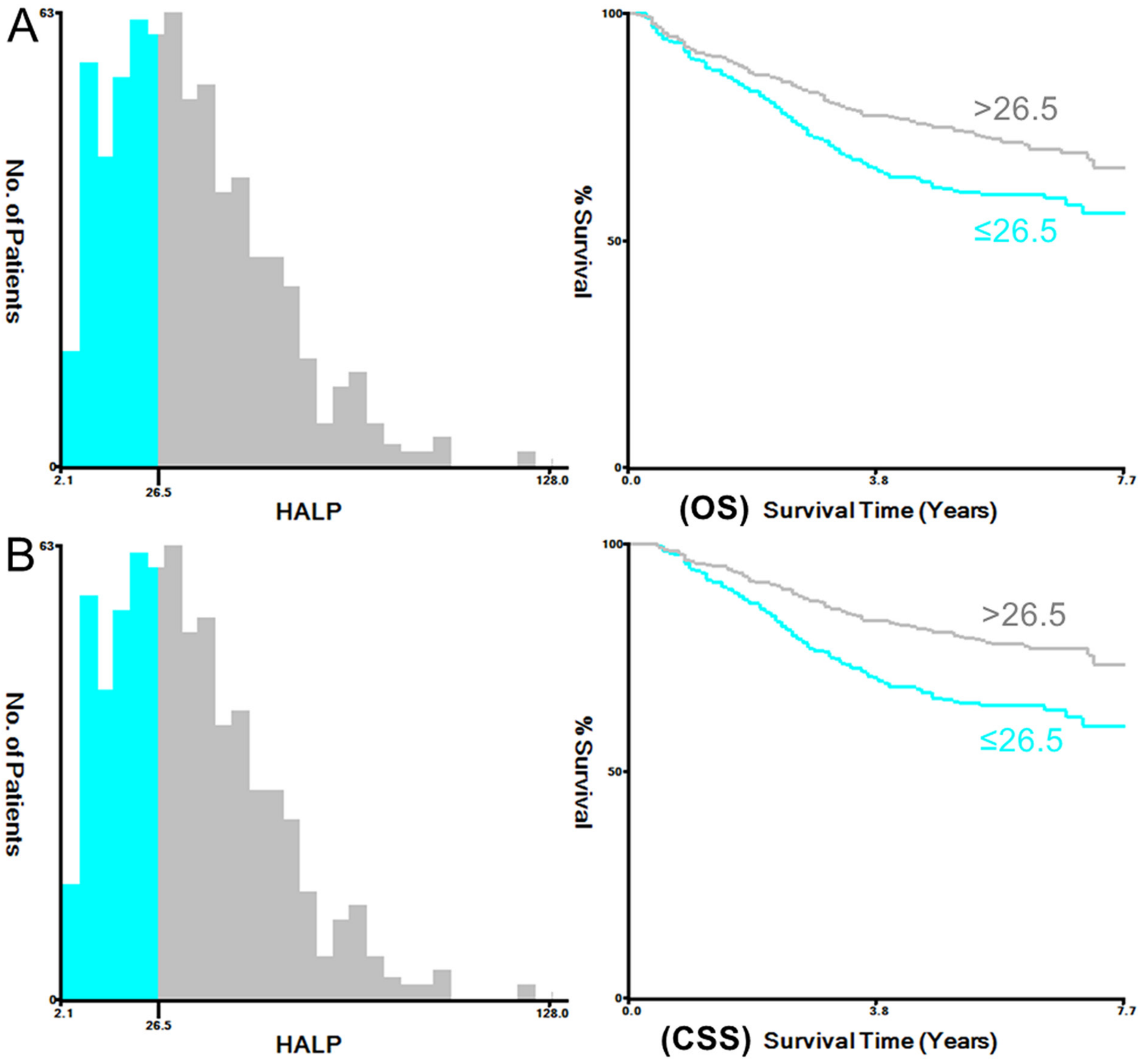

Supplementary Figure S1: X-tile analyses for A. OS and B. CSS, according to HALP.

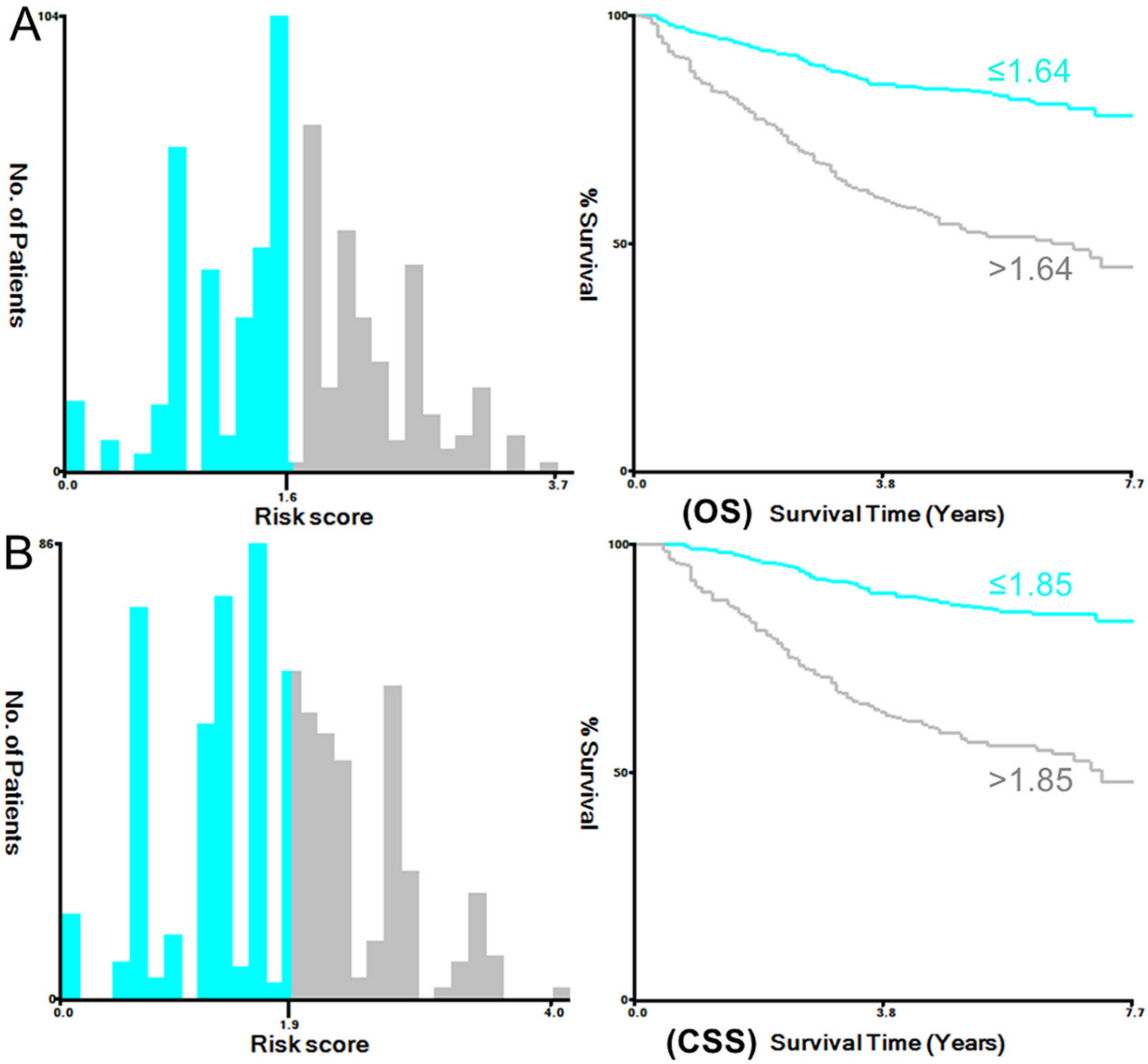

Supplementary Figure S2: X-tile analyses for A. OS and B. CSS, according to risk score.
